# IL-17 signaling in skin repair: safeguarding metabolic adaptation of wound epithelial cells

**DOI:** 10.1038/s41392-022-01202-9

**Published:** 2022-10-08

**Authors:** Juan Wang, Xiaolei Ding

**Affiliations:** 1 Institute of Geriatrics, Affiliated Nantong Hospital of Shanghai University (The Sixth People’s Hospital of Nantong), School of Medicine, Shanghai University, 226011 Nantong, China; 2 Shanghai Engineering Research Center of Organ Repair, School of Medicine, Shanghai University, 200444 Shanghai, China

**Keywords:** Molecular medicine, Cell biology

In a recent paper published in *Science*, Konieczny et al. describe that IL-17A produced by RORγt + γδ T cells governs wound epithelial cell hypoxic adaptation, which is required for cell migratory activity and efficient re-epithelization.^1^

**Fig. 1:**
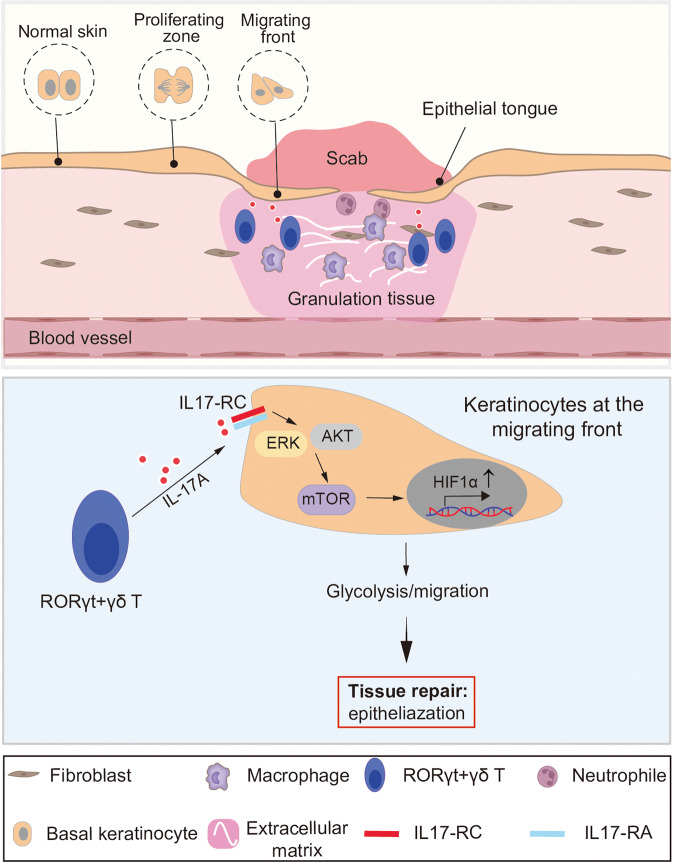
IL-17A signaling promotes epithelization by shaping wound epithelial cell metabolism. (Top) The scheme illustrates cellular networks involved in wound healing progression. Upon injury, immune cells, including neutrophils, macrophages, and lymphocytes, initiate an inflammatory response, which orchestrates wound healing progression. Fibroblasts become myofibroblasts, contributing to granulation formation. The activated keratinocytes at the wound edge migrate over the freshly formed granulation, re-epithelizing and restoring skin barrier function. Konieczny and colleagues report that RORγt + γδ T-cell-derived IL-17A regulates wound epithelization. (Bottom) RORγt + γδ T-cell-derived IL-17A signaling is required for the glycolytic metabolism and migration of wound epithelial tongue cells. IL-17A binds to its receptor IL-17-RC and induces HIF1α expression through ERK/AKT activated mTOR signaling. IL-17A-HIF1α signaling axis-mediated glycolytic metabolism is essential for the migratory activity of wound keratinocytes, thereby regulating wound epithelization

Skin wound healing is a highly dynamic and well-organized process that involves a series of distinct and overlapping phases: inflammation, tissue growth, and remolding (Fig. 1). A complex network of cytokines and growth factors orchestrates wound healing procedures by mediating cellular interactions. In particular, inflammatory signals determine many aspects of tissue repair and regenerative processes, and their dysregulation frequently results in aberrant inflammatory responses and subsequent failure in re-epithelization. Both are key features of slow or non-healing skin ulcers, which are a growing health concern worldwide.^2^ Understanding the mechanisms underlying wound healing is critical for developing novel therapies for wound healing disorders. Konieczny et al. uncover a novel immune-epithelial crosstalk mechanism in skin repair that regulates wound epithelial cell metabolic adaptation, migration, and timely epithelization (Fig. 1).

Skin lymphocytes function as sentinels, allowing for a rapid response to tissue damage.^3^ To obtain a comprehensive picture of the inflammatory response of lymphatic cells in wound healing, Konieczny and colleagues performed single-cell transcript analyses with lymphocytes (CD45^+^CD90^+^) isolated from wound tissues of healthy mice on days 3 and 5 following injury. Bioinformatics analysis reveals that interleukin 17 A (IL-17A) is one of the significantly upregulated cytokines in wound tissues. IL-17A is produced mainly from Th17 cells. Retinoic acid-related orphan receptor (RORγt) is a master regulator of Th17 cell differentiation and IL-17A expression. Immunostaining analysis demonstrates the enrichment of RORγt+ cells in both human and mouse wound tissues. Their expansion is likely via the proliferation of skin-resident cells rather than infiltration. Using a mouse model expressing green fluorescent protein (GFP) under the control regulatory elements of the *Rorgt* gene (which encodes RORγt), simultaneously deleting its expression (GFP-KI), they show that RORγt deficiency or treatment with a RORγt inhibitor diminishes the length of wound epithelial tongue and delays wound closure. In a detailed analysis that combines multiparametric flow cytometry and transgenic mouse models, the authors further underscore that a subset of RORγt + γδ T cells dictates wound epithelization and is the major source of IL-17A. Intriguingly, recombinant IL-17A administration can rescue the compromised epithelization in GFP-KI mice, implying that RORγt + γδ T-cell-derived IL-17A is required for wound epithelization (Fig. 1).

IL-17A signals to specific cells through binding to its receptors. Spatial transcriptomics (ST) analyses show that genes encoding IL-17 receptors A and C (*IL17ra* and *IL17rc*) are strongly upregulated in wound edge epithelial cells. The authors then generated epidermal-specific *IL17rc* deficient mice (*IL17rc*^EKO^). Interestingly, *IL17rc*^EKO^ wounds exhibit impaired epithelization, as seen in GFP-KI mice, further implicating a direct role for IL-17 signaling in wound epithelization (Fig. 1).

To gain insight into the molecular mechanisms that IL-17A signaling facilities wound epithelization, the authors returned to the ST and RNA-seq data to identify regulators that are potentially implicated in the wound healing impairment observed in IL-17A signaling-deficient mice. In addition to the altered IL-17 signaling, gene signatures for hypoxia-induced factor 1α (HIF1α) and mechanistic target of rapamycin (mTOR) signaling pathways are enriched. In line, compared with controls HIF1α expression at the wound edge is significantly decreased in both GFP-KI and *IL17rc*^EKO^ mice. HIF1α plays a crucial role in glycolysis in response to hypoxia.^4^ HIF1α-mediated glycolysis is essential for efficient epithelization, as inhibiting glycolysis either pharmaceutically or genetically by deleting HIF1α expression delays wound epithelization. Using in vitro skin epithelial organoids and in vivo mouse models, the authors further show that IL-17A signaling enhances HIF1α expression through promoting HIF1α mRNA transcription rather than stabilizing HIF1α protein. The combination of hypoxia and IL-17 signaling can induce HIF1α expression sufficiently, triggering the gene expression program involved in glycolysis and the consequent cell migratory activity. However, when IL-17 signaling is disrupted, hypoxia, particularly under chronic conditions, appears insufficient to ensure HIF1α expression at the wound edge, resulting in impaired epithelization. IL-17A-HIF1α signaling axis enables epithelial tongue cells to rapidly produce energy and subsequently carry out their migratory activity, thereby enhancing wound epithelization (Fig. 1). Thus, Konieczny et al. have challenged the notion that HIF1α-mediated glycolytic metabolism is driven by hypoxia by proposing that IL-17 signaling safeguards epithelial cell hypoxic adaptation through promoting HIF1α expression.

Immunostaining shows that the level of phosphorylated ribosomal protein S6 is significantly lower in GFP-KI mouse wounds compared to controls, indicating that the activation of mTOR signaling is disrupted. mTOR may drive HIF1α expression via multiple mechanisms at both transcriptional and translational level.^4^ Using in vitro cultured cells and in vivo mouse models, Konieczny et al. demonstrate that IL-17A binds to its receptor and signals to mTOR via ERK/AKT kinases. The activated mTOR signaling is essential for HIF1α expression, particularly under long-term hypoxia conditions.

The data of Konieczny et al. are likely to be relevant in human settings and also raise several interesting questions for future study. Disturbed cell migration and angiogenic responses are the major pathogenic mechanisms underlying diabetic foot ulcers,^2^ and diabetic wounds are characterized by reduced mTOR signaling.^5^ Hence, it would be exciting to explore whether the compromised interplay described here predisposes to the development of chronic- unhealing wounds. Furthermore, the findings suggest a potential beneficial effect of IL-17A signaling in wound healing, and manipulating IL-17A signaling may have therapeutic implications in treating chronic wounds. However, any consideration of IL-17A administration for enhancing tissue repair should be carefully examined for its detrimental effects, such as the pro-inflammatory role in autoimmune disease. In addition, it would be important to determine the regulation of IL-17 signaling on the other types of wound cells, e.g., macrophages, which have been recently demonstrated to be characterized by a high glycolytic program at the early phase of repair.^6^

Overall, the study by Konieczny and colleagues suggests a model that wound epithelial cell migratory activity depends on HIF1α-mediated glycolytic metabolism, which is maintained by a subset of RORγt + γδ T-cell-derived IL-17A signaling. Modulating the IL-17A-mTOR-HIF1α signaling axis may represent a novel clinical therapeutic strategy for wound healing disorders.
